# Supplementary material for: Eating in a losing cause: limited benefit of modified macronutrient consumption following infection in the oriental cockroach Blatta orientalis
Source: BMC Ecol Evol. 2022 May 18;22:67. doi: 10.1186/s12862-022-02007-8 (PMC9118584; doi:10.1186/s12862-022-02007-8)
Supplement: Supplementary file 13 — Additional file 13. Supplementary File (Supplementary Tables andFigures). [file 12862_2022_2007_MOESM13_ESM.docx]

**Sample preparation**

Hemolymph of the two groups of insects (fed with carbohydrate or protein-rich diets) was extracted as described in the main text. Four and six biological replicates per group were prepared for the first and second bacterial inhibition assays, respectively. Each group consisted of a pool of hemolymph from ten individuals, prepared for proteomic analysis by mass spectrometry. The protein content of hemolymph was determined using the BCA Protein Assay Kit (Pierce™, Thermo Scientific™, Germany). In total, 18 µl of denaturation buffer (Urea 6 M, Thiourea 2 M and HEPES 10 mM) were added to 2 µl of hemolymph samples (this corresponds approximately to 50-60 µg of total protein) and used for in-solution protein digestion as described previously (Sury *et al.* 2015). Protein mixtures solubilized in denaturation buffer were reduced with 10 mM DTT and then alkylated with 55 mM iodoacetamide for 30 min each. A pre-digestion with lysyl endopeptidase (LysC, Wako, Japan) was carried out overnight using a proportion of 1 µg enzyme per every 50 µg of protein sample. After pre-digestion with LysC, the samples were diluted fourfold with 50 mM ammonium bicarbonate and subjected to overnight trypsin digestion using 1 µg of sequencing-grade modified trypsin (Promega, Madison, USA). All in-solution protein digestion steps were performed at room temperature. The reactions were stopped by adding an equal volume of Buffer A* (5 % acetonitrile, 3 % trifluoroacetic acid). Samples were then desalted and stored using self-made StageTips (Rappsilber *et al.* 2007).

**Liquid chromatography-mass spectrometry (LC-MS)**

Peptides were reconstituted in 15 μl of 0.1% TFA, 5 % acetonitrile and 2 µl were analysed by a reversed-phase capillary nano liquid chromatography system (Ultimate 3000, Thermo Scientific, USA) connected to an Orbitrap Velos mass spectrometer (Thermo Scientific). LC separations were performed on a capillary column (Acclaim PepMap100 C18, 2 μm, 100 Å, 75 μm i.d. × 25 cm, Thermo Scientific) at an eluent flow rate of 300 nl/min using a gradient of 3–50% mobile phase B in 50 min. Mobile phase A contained 0.1% formic acid in water, and mobile phase B contained 0.1% formic acid in acetonitrile. Mass spectra were acquired in a data-dependent mode utilizing a single MS survey scan (m/z 350-1500) with a resolution of 60,000 in the Orbitrap, and MS/MS scans by collision-induced dissociation (CID) of the 20 most intense precursor ions in the linear trap quadrupole.

**Proteomic database preparation by *de novo* transcriptome sequencing**

Whole bodies of 8 adult cockroaches were injected through the cuticle with 5 x 10^6^ CFU/g of a cocktail of heat-killed microbes (*P. entomophila, B. thuringiensis, S. cerevisiae*) and, as a control, an equal number of cockroaches were injected with Ringer’s solution. Both immune-challenged and control individuals were used for total RNA extraction, described briefly as follows. Individuals were cut into 4-6 pieces with sterile scissors and RNA was extracted separately before being pooled. Each piece was suspended in pre-cooled Trizol (Thermo Fisher Scientific) and homogenized using a homogenizer (FastPrep™-24, MP Biomedicals) with a 5mm stainless steel bead (Qiagen). Recovery of RNA was achieved following manufacturer’s instructions, using chloroform extraction and isopropanol precipitation, and re-dissolved in RNA storage solution (Ambion). Subsequently, samples were incubated with 2 units of TurboDNase (Ambion) for 30 min at 37 °C and RNA was purified using RNAeasy Mini kit (Qiagen) according to manufacturer's instructions. Quantity and quality of RNA were determined by Qubit and Bioanalyzer 2100. Equal amounts of total RNA from 4 individual extractions were pooled together to form 4 libraries. Subsequently, the mRNA libraries were enriched and prepared using a NEXTflexTM Rapid Directional mRNA-seq Kit protocol (Bioo Scientific). The prepared libraries were sequenced on an Illumina NextSeq500/550 platform at the Berlin Center for Genomics in Biodiversity Research (BeGenDiv).

The raw data were analyzed as described elsewhere (He et al. 2018). Briefly, reads were trimmed and filtered using Trimmomatic, as incorporated in Trinity (v2.2.0) (Grabherr et al. 2011). Paired-end reads were assembled using Trinity with default parameters (Kmer size: 25) on a local server. The transcriptome was annotated following the guidelines of Trinotate (https://trinotate.github.io/). Protein domains, signal peptides, and transmembrane domains were determined by HMMER (v3.1b2) against the pfam database (Finn et al. 2011), SignalP v4.0 (Petersen et al. 2011), and TMHMM 2.0 (Krogh et al. 2001), respectively. Homology searches, predictions and domain identifications were performed locally and subsequently integrated into SQLite database with SQLite v3.11.0 at an e-value threshold of 1e^-03^. The highest expressed isoforms from each contig were filtered for following proteomic data analysis.

**Proteomic data analysis**

Identification and label-free quantification of proteins was performed using the freely available software suit MaxQuant (v1.6.0.1) with implemented Andromeda search engine (Cox & Mann 2008; Cox *et al.* 2011; Tyanova *et al.* 2015). Raw data were matched against an in-house protein database of *B. orientalis* created by *de novo* transcriptome sequencing (see above). Trypsin was selected as enzyme and a maximum of two missed cleavages was allowed. Cysteine carbamidomethylation was set as a fixed modification. Methionine oxidation and acetylation (protein N-terminus) were allowed as variable modifications. The ’match between runs’ option used a 0.7 min match time window and a 20 min alignment time window. The option to report iBAQ values was activated (insert PMID: 21593866). The minimum peptide length was set to 7 amino acids and the false discovery rate (FDR) for peptide and protein identification was set to 0.01.

**Proteomic data processing and statistical analysis**

Data processing and statistical analysis was performed with the PERSEUS software (v1.6.0.2) (Tyanova et al. 2016; Tyanova & Cox 2018). Proteins identified from the contaminant database, reverse hits and proteins only identified by site were removed as well as proteins with less than three valid values. For the remaining 387 proteins imputation of missing values was performed (width 0.3, down shift 1.8). Significant changes in protein abundance between the two treatments were calculated by a student's t-test using permutation-based FDR of 0.05. Only proteins with significant abundance changes (q<0.05) were considered. In addition, a minimum 2-fold change in protein abundance was set as a threshold.

After peptides from the LC-MS/MS analysis were matched to our transcriptome-derived predicted protein database, we ascertained the putative functions of proteins by querying the annotated functional database associated with our transcriptome-derived predicted-protein database. The functional annotation of the majority of these proteins was derived from SwissProt queries using BLAST (blastp) and proteins that could not be identified using blastp were assigned using HMMER search results from the annotated functional database. Proteins that could not be annotated with Trinotate suit tools (blastp, HMMER) were assigned “unknown” function.

Supplementary Tab. 1: Ingredients of artificial diets and vitamin mix composition. Ingredients were dissolved in 1 % agar solution in a 1:6 w/v ratio.

| **Ingredient** | **Amount** |
| --- | --- |
| carbohydrate (sucrose) | 35 % or 7 % or 21 % |
| protein (casein, peptone and albumin from eggs in a 3:1:1 ratio) | 35 % or 7 % or 21 % |
| linoleic acid | 0.5 % |
| cholesterol | 0.5 % |
| Wesson’s salt mixture | 2.5 % |
| ascorbate | 0.3 % |
| cellulose | 54 % |
| **Vitamin mix:** | 0.2 % |
| - thiamine | 0.075 g |
| - riboflavin | 0.075 g |
| - nicotinic acid | 0.3 g |
| - pyridoxine | 0.075 g |
| - folic acid | 0.075 g |
| - meso-inositol | 0.75 g |
| - calcium pantothenate | 0.15 g |
| - p-aminobenzoic acid | 0.075 g |
| - choline chloride | 3.75 g |
| - biotin | 0.003 g |

Supplementary Tab. 2: Median and mean P:C ratios, amounts of P and C eaten as well as total consumption differences (in mg) for each treatment in the food choice experiment. Significance of test of normality using the ksnormal function of the wrappedtools package v0.3.10 is also shown.

| **Parameter** | **Day** | **Treatment** | **P value** | **Median** | **Mean** | **Comment** |
| --- | --- | --- | --- | --- | --- | --- |
| Ratio | 0 | All together | 0.001 | 0.236 | 0.282 | Prior to treatment |
| Ratio | 1 | Control | 0.128 | 0.228 | 0.252 |  |
| Ratio | 1 | Ringers | 0.836 | 0.869 | 0.943 |  |
| Ratio | 1 | Low | 0.224 | 0.963 | 1.063 |  |
| Ratio | 1 | High | 0.285 | 1.232 | 1.299 |  |
| P proportion | 0 | All together | 0.000 | 0.080 | 0.090 | Prior to treatment |
| P proportion | 1 | Control | 0.182 | 0.078 | 0.084 |  |
| P proportion | 1 | Ringers | 0.549 | 0.195 | 0.190 |  |
| P proportion | 1 | Low | 0.662 | 0.206 | 0.187 |  |
| P proportion | 1 | High | 0.708 | 0.232 | 0.228 |  |
| C proportion | 0 | All together | 0.000 | 0.340 | 0.330 | Prior to treatment |
| C proportion | 1 | Control | 0.182 | 0.342 | 0.336 |  |
| C proportion | 1 | Ringers | 0.549 | 0.225 | 0.230 |  |
| C proportion | 1 | Low | 0.662 | 0.214 | 0.233 |  |
| C proportion | 1 | High | 0.708 | 0.188 | 0.192 |  |
| C eaten | 0 | All together | 0.744 | 2.209 | 2.241 | Prior to treatment |
| C eaten | 1 | Control | 0.777 | 2.497 | 2.853 |  |
| C eaten | 1 | Ringers | 0.348 | 0.169 | 0.217 |  |
| C eaten | 1 | Low | 0.348 | 0.185 | 0.232 |  |
| C eaten | 1 | High | 0.812 | 0.150 | 0.171 |  |
| P eaten | 0 | All together | 0.804 | 0.561 | 0.579 | Prior to treatment |
| P eaten | 1 | Control | 0.740 | 0.669 | 0.674 |  |
| P eaten | 1 | Ringers | 0.453 | 0.204 | 0.204 |  |
| P eaten | 1 | Low | 0.410 | 0.219 | 0.190 |  |
| P eaten | 1 | High | 0.127 | 0.256 | 0.215 |  |
| Eaten total | 0 | All together | 0.887 | 6.613 | 6.714 | Prior to treatment |
| Eaten total | 1 | Control | 0.850 | 7.480 | 8.398 |  |
| Eaten total | 1 | Ringers | 0.281 | 0.815 | 1.003 |  |
| Eaten total | 1 | Low | 0.966 | 0.945 | 1.004 |  |
| Eaten total | 1 | High | 0.685 | 0.975 | 0.919 |  |

Supplementary Tab. 3: GLMM post-hoc comparisons for the proportion of protein and carbohydrate chosen (mean values from day 1 p.i. are shown).

| **Comparison** | **Protein proportion chosen day 1 post infection** | **z** | **p** |
| --- | --- | --- | --- |
| high vs. low | 0.228 vs 0.187 | -6.285 | < 0.001 |
| high vs. unmanipulated | 0.228 vs 0.084 | -13.062 | < 0.001 |
| high vs. wounded | 0.228 vs 0.190 | -4.786 | < 0.001 |
| low vs. unmanipulated | 0.187 vs 0.084 | -5.322 | < 0.001 |
| low vs. wounded | 0.187 vs 0.190 | 1.038 | > 0.1 |
| wounded vs. unmanipulated | 0.190 vs 0.084 | -6.132 | < 0.001 |
|  |  |  |  |
| **Comparison** | **Carbohydrate proportion chosen day 1 post infection** | **z** | **p** |
| high vs. low | 0.192 vs 0.233 | 4.893 | < 0.001 |
| high vs. unmanipulated | 0.192 vs 0.336 | 9.989 | < 0.001 |
| high vs. wounded | 0.192 vs 0.230 | 4.107 | < 0.001 |
| low vs. unmanipulated | 0.233 vs 0.336 | 3.736 | < 0.01 |
| low vs. wounded | 0.233 vs 0.230 | -0.494 | > 0.1 |
| wounded vs. unmanipulated | 0.230 vs 0.336 | 4.204 | < 0.001 |

Supplementary Tab. 4: Cox proportional hazard post-hoc comparisons for the survival on enforced diet after infection with Bonferroni correction for multiple testing. First letter = Treatment: B = bacteria infection, W = wounding (Ringer’s solution), U = unmanipulated; second letter = diet: P = protein, C = carbohydrate, E = equal

**Estimate** **Std. Error** **z** **p**

BP - BC == 0 -1.190e-01 1.646e-01 -0.723 > 0.1

BE - BC == 0 -4.224e-01 1.687e-01 -2.504 > 0.1

UC - BC == 0 -3.360e+00 7.168e-01 -4.687 < 0.001

UP - BC == 0 -3.360e+00 7.168e-01 -4.687 < 0.001

UE - BC == 0 -3.360e+00 7.168e-01 -4.687 < 0.001

WC - BC == 0 -3.360e+00 7.168e-01 -4.687 < 0.001

WP - BC == 0 -3.360e+00 7.168e-01 -4.687 < 0.001

WE - BC == 0 -2.954e+00 5.892e-01 -5.013 < 0.001

BE - BP == 0 -3.034e-01 1.719e-01 -1.765 > 0.1

UC - BP == 0 -3.241e+00 7.174e-01 -4.517 < 0.001

UP - BP == 0 -3.241e+00 7.174e-01 -4.517 < 0.001

UE - BP == 0 -3.241e+00 7.174e-01 -4.517 < 0.001

WC - BP == 0 -3.241e+00 7.174e-01 -4.517 < 0.001

WP - BP == 0 -3.241e+00 7.174e-01 -4.517 < 0.001

WE - BP == 0 -2.835e+00 5.900e-01 -4.805 < 0.001

UC - BE == 0 -2.937e+00 7.183e-01 -4.090 < 0.01

UP - BE == 0 -2.937e+00 7.183e-01 -4.090 < 0.01

UE - BE == 0 -2.937e+00 7.183e-01 -4.090 < 0.01

WC - BE == 0 -2.937e+00 7.183e-01 -4.090 < 0.01

WP - BE == 0 -2.937e+00 7.183e-01 -4.090 < 0.01

WE - BE == 0 -2.531e+00 5.910e-01 -4.283 < 0.001

UP - UC == 0 -5.773e-15 1.000e+00 0.000 > 0.1

UE - UC == 0 1.776e-15 1.000e+00 0.000 > 0.1

WC - UC == 0 -2.354e-14 1.000e+00 0.000 > 0.1

WP - UC == 0 -1.998e-14 1.000e+00 0.000 > 0.1

WE - UC == 0 4.061e-01 9.129e-01 0.445 > 0.1

UE - UP == 0 7.550e-15 1.000e+00 0.000 > 0.1

WC - UP == 0 -1.776e-14 1.000e+00 0.000 > 0.1

WP - UP == 0 -1.421e-14 1.000e+00 0.000 > 0.1

WE - UP == 0 4.061e-01 9.129e-01 0.445 > 0.1

WC - UE == 0 -2.531e-14 1.000e+00 0.000 > 0.1

WP - UE == 0 -2.176e-14 1.000e+00 0.000 > 0.1

WE - UE == 0 4.061e-01 9.129e-01 0.445 > 0.1

WP - WC == 0 3.553e-15 1.000e+00 0.000 > 0.1

WE - WC == 0 4.061e-01 9.129e-01 0.445 > 0.1

WE - WP == 0 4.061e-01 9.129e-01 0.445 > 0.1

Supplementary Tab. 5: Cox proportional hazard post-hoc comparisons for the survival on enforced diet after infection with correction for multiple testing using the false discovery rate approach. First letter = Treatment: B = bacteria infection, W = wounding (Ringer’s solution), U = unmanipulated; second letter = diet: P = protein, C = carbohydrate, E = equal

**Estimate Std. Error z p**

BP - BC == 0 -1.190e-01 1.646e-01 -0.723 > 0.1

BE - BC == 0 -4.224e-01 1.687e-01 -2.504 < 0.05

UC - BC == 0 -3.360e+00 7.168e-01 -4.687 < 0.001

UP - BC == 0 -3.360e+00 7.168e-01 -4.687 < 0.001

UE - BC == 0 -3.360e+00 7.168e-01 -4.687 < 0.001

WC - BC == 0 -3.360e+00 7.168e-01 -4.687 < 0.001

WP - BC == 0 -3.360e+00 7.168e-01 -4.687 < 0.001

WE - BC == 0 -2.954e+00 5.892e-01 -5.013 < 0.001

BE - BP == 0 -3.034e-01 1.719e-01 -1.765 > 0.1

UC - BP == 0 -3.241e+00 7.174e-01 -4.517 < 0.001

UP - BP == 0 -3.241e+00 7.174e-01 -4.517 < 0.001

UE - BP == 0 -3.241e+00 7.174e-01 -4.517 < 0.001

WC - BP == 0 -3.241e+00 7.174e-01 -4.517 < 0.001

WP - BP == 0 -3.241e+00 7.174e-01 -4.517 < 0.001

WE - BP == 0 -2.835e+00 5.900e-01 -4.805 < 0.001

UC - BE == 0 -2.937e+00 7.183e-01 -4.090 < 0.001

UP - BE == 0 -2.937e+00 7.183e-01 -4.090 < 0.001

UE - BE == 0 -2.937e+00 7.183e-01 -4.090 < 0.001

WC - BE == 0 -2.937e+00 7.183e-01 -4.090 < 0.001

WP - BE == 0 -2.937e+00 7.183e-01 -4.090 < 0.001

WE - BE == 0 -2.531e+00 5.910e-01 -4.283 < 0.001

UP - UC == 0 -5.773e-15 1.000e+00 0.000 > 0.1

UE - UC == 0 1.776e-15 1.000e+00 0.000 > 0.1

WC - UC == 0 -2.354e-14 1.000e+00 0.000 > 0.1

WP - UC == 0 -1.998e-14 1.000e+00 0.000 > 0.1

WE - UC == 0 4.061e-01 9.129e-01 0.445 > 0.1

UE - UP == 0 7.550e-15 1.000e+00 0.000 > 0.1

WC - UP == 0 -1.776e-14 1.000e+00 0.000 > 0.1

WP - UP == 0 -1.421e-14 1.000e+00 0.000 > 0.1

WE - UP == 0 4.061e-01 9.129e-01 0.445 > 0.1

WC - UE == 0 -2.531e-14 1.000e+00 0.000 > 0.1

WP - UE == 0 -2.176e-14 1.000e+00 0.000 > 0.1

WE - UE == 0 4.061e-01 9.129e-01 0.445 > 0.1

WP - WC == 0 3.553e-15 1.000e+00 0.000 > 0.1

WE - WC == 0 4.061e-01 9.129e-01 0.445 > 0.1

WE - WP == 0 4.061e-01 9.129e-01 0.445 > 0.1

Supplementary Tab. 6: Statistic of the “Bacteria growth inhibition assay”. B = infected with *P. entomophila*, W = wounded (Ringer-injected), U = unmanipulated**,** Neg = negative control (only medium), Pos = positive control (medium and bacteria), P = high protein diet, C = high carbohydrate diet, E = equal diet.

| **.y.** | **Group 1** | **Group 2** | **n1** | **n2** | **statistic** | **df** | **p** | **p.adj fdr** | **p.adj bonferroni** |
| --- | --- | --- | --- | --- | --- | --- | --- | --- | --- |
| auc_e | BC | BE | 4 | 4 | 0.080880924 | 5.491410694 | 0.938 | 0.951 | 1 |
| auc_e | BC | BP | 4 | 4 | 0.902241916 | 3.211628719 | 0.429 | 0.644 | 1 |
| auc_e | BC | Neg | 4 | 4 | 1.502219441 | 3.001209427 | 0.23 | 0.469 | 1 |
| auc_e | BC | Pos | 4 | 4 | -6.106839039 | 3.120252341 | 0.008 | 0.047 | 0.433 |
| auc_e | BC | UC | 4 | 4 | -0.983503944 | 5.774234298 | 0.365 | 0.615 | 1 |
| auc_e | BC | UE | 4 | 4 | -0.659529589 | 5.725735153 | 0.535 | 0.736 | 1 |
| auc_e | BC | UP | 4 | 4 | -0.898803959 | 3.940325784 | 0.42 | 0.644 | 1 |
| auc_e | BC | WC | 4 | 4 | -0.155595128 | 5.985501733 | 0.881 | 0.925 | 1 |
| auc_e | BC | WE | 4 | 4 | -0.64172661 | 4.704222953 | 0.551 | 0.739 | 1 |
| auc_e | BC | WP | 4 | 4 | 0.263489125 | 3.380459174 | 0.807 | 0.902 | 1 |
| auc_e | BE | BP | 4 | 4 | 1.084574209 | 3.395547042 | 0.349 | 0.615 | 1 |
| auc_e | BE | Neg | 4 | 4 | 1.919659606 | 3.002267575 | 0.151 | 0.371 | 1 |
| auc_e | BE | Pos | 4 | 4 | -8.425592922 | 3.225235534 | 0.003 | 0.024 | 0.146 |
| auc_e | BE | UC | 4 | 4 | -1.249940664 | 5.923797437 | 0.258 | 0.493 | 1 |
| auc_e | BE | UE | 4 | 4 | -0.798549027 | 4.836338376 | 0.462 | 0.669 | 1 |
| auc_e | BE | UP | 4 | 4 | -1.282512439 | 4.657957279 | 0.26 | 0.493 | 1 |
| auc_e | BE | WC | 4 | 4 | -0.262521561 | 5.622841065 | 0.802 | 0.902 | 1 |
| auc_e | BE | WE | 4 | 4 | -0.908479052 | 5.613437209 | 0.401 | 0.644 | 1 |
| auc_e | BE | WP | 4 | 4 | 0.222077879 | 3.706158729 | 0.836 | 0.902 | 1 |
| auc_e | BP | Neg | 4 | 4 | 3.100588479 | 3.03424528 | 0.052 | 0.163 | 1 |
| auc_e | BP | Pos | 4 | 4 | -30.11400743 | 5.576099868 | 0.000000218 | 0.000012 | 0.000012 |
| auc_e | BP | UC | 4 | 4 | -2.606732578 | 3.315465958 | 0.072 | 0.209 | 1 |
| auc_e | BP | UE | 4 | 4 | -1.562186301 | 3.135727282 | 0.212 | 0.448 | 1 |
| auc_e | BP | UP | 4 | 4 | -4.259992135 | 4.257304728 | 0.011 | 0.057 | 0.627 |
| auc_e | BP | WC | 4 | 4 | -1.167521516 | 3.233474791 | 0.322 | 0.59 | 1 |
| auc_e | BP | WE | 4 | 4 | -2.806361027 | 3.671351069 | 0.054 | 0.163 | 1 |
| auc_e | BP | WP | 4 | 4 | -2.054220583 | 5.545072332 | 0.09 | 0.246 | 1 |
| auc_e | Neg | Pos | 4 | 4 | -53.89791923 | 3.060314092 | 0.0000117 | 0.000214 | 0.000644 |
| auc_e | Neg | UC | 4 | 4 | -3.388034328 | 3.0018056 | 0.043 | 0.157 | 1 |
| auc_e | Neg | UE | 4 | 4 | -2.047421584 | 3.000775092 | 0.133 | 0.348 | 1 |
| auc_e | Neg | UP | 4 | 4 | -6.158132086 | 3.007522616 | 0.009 | 0.047 | 0.47 |
| auc_e | Neg | WC | 4 | 4 | -1.803691804 | 3.001334636 | 0.169 | 0.381 | 1 |
| auc_e | Neg | WE | 4 | 4 | -4.006587369 | 3.003881105 | 0.028 | 0.109 | 1 |
| auc_e | Neg | WP | 4 | 4 | -4.869472537 | 3.018995972 | 0.016 | 0.075 | 0.896 |
| auc_e | Pos | UC | 4 | 4 | 5.895681135 | 3.179440842 | 0.008 | 0.047 | 0.454 |
| auc_e | Pos | UE | 4 | 4 | 4.066699176 | 3.077085029 | 0.026 | 0.108 | 1 |
| auc_e | Pos | UP | 4 | 4 | 12.22720234 | 3.736810104 | 0.000378 | 0.005 | 0.021 |
| auc_e | Pos | WC | 4 | 4 | 6.185438973 | 3.13269016 | 0.007 | 0.047 | 0.412 |
| auc_e | Pos | WE | 4 | 4 | 9.433487324 | 3.384458954 | 0.002 | 0.017 | 0.084 |
| auc_e | Pos | WP | 4 | 4 | 22.25592138 | 4.719044906 | 0.00000582 | 0.00016 | 0.00032 |
| auc_e | UC | UE | 4 | 4 | 0.144354281 | 5.174857011 | 0.891 | 0.925 | 1 |
| auc_e | UC | UP | 4 | 4 | 0.331957117 | 4.361687519 | 0.755 | 0.884 | 1 |
| auc_e | UC | WC | 4 | 4 | 0.841233818 | 5.868009274 | 0.433 | 0.644 | 1 |
| auc_e | UC | WE | 4 | 4 | 0.540999519 | 5.294708832 | 0.61 | 0.784 | 1 |
| auc_e | UC | WP | 4 | 4 | 1.801234385 | 3.565198483 | 0.155 | 0.371 | 1 |
| auc_e | UE | UP | 4 | 4 | 0.066283851 | 3.611714249 | 0.951 | 0.951 | 1 |
| auc_e | UE | WC | 4 | 4 | 0.535171691 | 5.605684339 | 0.613 | 0.784 | 1 |
| auc_e | UE | WE | 4 | 4 | 0.234160313 | 4.152295979 | 0.826 | 0.902 | 1 |
| auc_e | UE | WP | 4 | 4 | 1.041349548 | 3.244408395 | 0.369 | 0.615 | 1 |
| auc_e | UP | WC | 4 | 4 | 0.729514349 | 4.032012682 | 0.506 | 0.714 | 1 |
| auc_e | UP | WE | 4 | 4 | 0.339615502 | 5.444795492 | 0.747 | 0.884 | 1 |
| auc_e | UP | WP | 4 | 4 | 2.6176505 | 5.053942921 | 0.047 | 0.161 | 1 |
| auc_e | WC | WE | 4 | 4 | -0.471320307 | 4.845092119 | 0.658 | 0.804 | 1 |
| auc_e | WC | WP | 4 | 4 | 0.494074074 | 3.419478389 | 0.651 | 0.804 | 1 |
| auc_e | WE | WP | 4 | 4 | 1.643293069 | 4.176740158 | 0.173 | 0.381 | 1 |

Supplementary Tab. 7: Results of the two-way ANOVA on the growthcurver results (R package growthcurver) for the combined antimicrobial activity assay of P_infected_ versus C_infected_ (N=10 replicates per treatment).

Df Sum Sq Mean Sq F value Pr(>F)

Treatment 1 382 382.4 0.564 0.463

Cohort 1 2 2.3 0.003 0.954

Treatment:Cohort 1 302 302.0 0.446 0.514

Residuals 16 10844 677.7

Supplementary Tab. 8. List of hemolymph proteins that show a significant change in relative abundance (> 2-fold) between a P-rich (blue) or C-rich (red) diet, in *B. orientalis* males challenged with 5.8 x 10^5^ *P. entomophila* CFUs, as measured by LC-MS analysis with label-free quantification.

| **Name** | **Main biological process** | **Gene ID** | **Blast e-value** | **Reference** |
| --- | --- | --- | --- | --- |
| Hexokinase type II | Carbohydrate metabolism (glycolysis) | HXK2_DROME | 2.81e-176 | Yanagawa 1978 |
| Carbonyl reductase 3 | NADPH-dependent reduction of biologically and pharmacologically active substrates including endogenous and xenobiotic carbonyl compounds | CBR3_MOUSE | 3.59e-84 | Hoffmann and Maser 2007 |
| L-galactose dehydrogenase | Unknown | GALDH_ARATH | 2.77e-75 |  |
| Tropomyosin | Calcium dependent regulation of muscle contraction | TPM_PERAM | 0.0 | Pomés *etal.* 2007 |
| Acyl-CoA-binding protein | Transport (Lipid binding);  Suppression of glucose-induced insulin secretion | ACBP_CHICK | 7.22e-33 | Færgeman *etal.* 2007;  Pasco and Léopold 2012 |
| Alpha-amylase | Carbohydrate metabolism | AMY_TENMO | 2.75e-89 | Terra and Ferreira 1994 |
| Proteasome subunit alpha type 3 | Protein degradation | PSA3_MOUSE | 2.4e-131 | Rivett 1993 |
| Hemolymph lipopolysaccharide-binding protein-like (2 isoforms) | Carbohydrate binding (probably foreign particles) | LPSBP_PERAM | 6.6e-48  5.83e-64 | Jomori and Natori 1991 |
| Superoxide dismutase | Extracellular superoxide metabolic process | SODE_CAEEL | 1.13e-47 | Felton and Summers 1995 |
| Ankyrin 1 | Unknown | ANK1_MOUSE | 7.01e-38 |  |
| Glutamine synthetase | Glutamate catabolic process;  Glutamine biosynthetic process;  Neurotransmitter receptor metabolic process | GLNA2_DROME | 0.0 | Smartt *etal.* 1998 |
| Adenylate kinase isoenzyme 1 | ATP metabolic process | KAD1_PIG | 3.31e-46 | Fujisawa *etal.* 2009 |
| Hexamerin | Nutrient reservoir activity (amino acid and energy storage) | HEXA_BLADI | 1.77e-83 | Burmester 1999 |


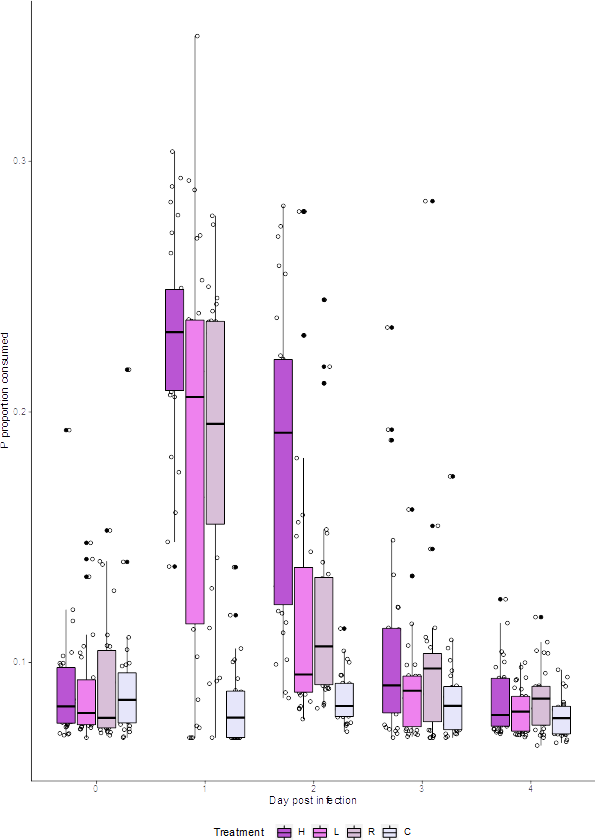
Supplementary Fig. 1: P proportion consumed. H = high infection, L = low infection, R = Ringers, C = Control.


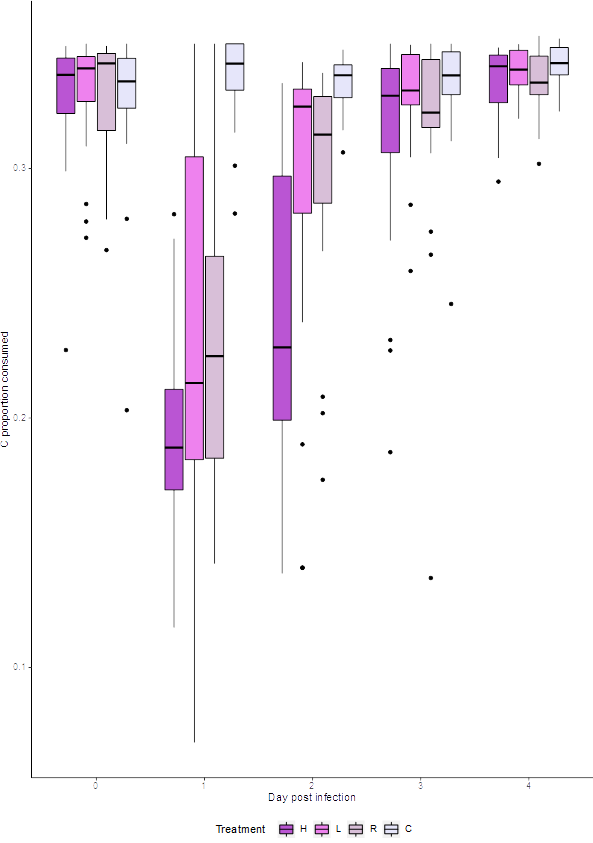
Supplementary Fig. 2: C proportion consumed. H = high infection, L = low infection, R = Ringers, C = Control.


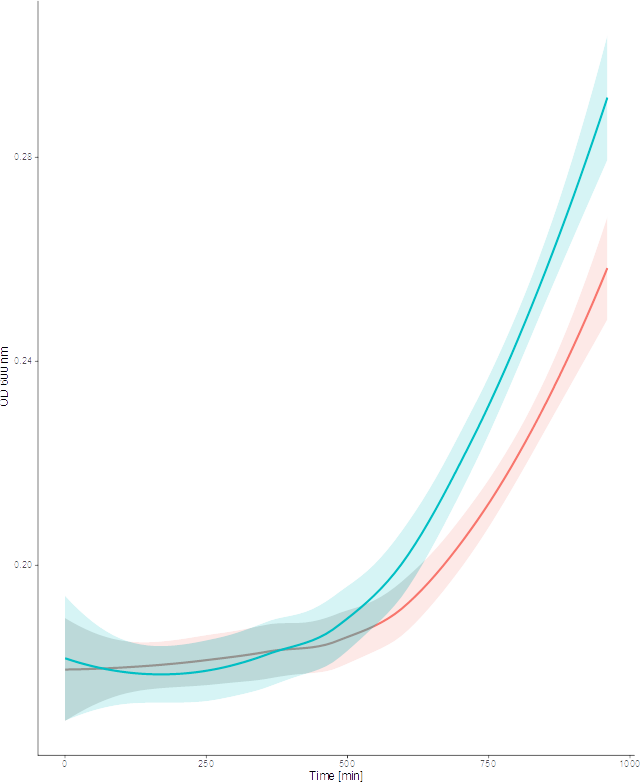


Supplementary Fig. 3: Growth curves for the combined antimicrobial activity assay. Blue = high C diet; Red = high P diet.

**References**

1. Cox, J. & Mann, M. (2008). MaxQuant enables high peptide identification rates, individualized p.p.b.-range mass accuracies and proteome-wide protein quantification. *Nat Biotechnol*, 26, 1367-1372.

2. Cox, J., Neuhauser, N., Michalski, A., Scheltema, R.A., Olsen, J.V. & Mann, M. (2011). Andromeda: a peptide search engine integrated into the MaxQuant environment. *Journal of proteome research*, 10, 1794-1805.

3. Finn, R.D., Clements, J. & Eddy, S.R. (2011). HMMER web server: interactive sequence similarity searching. *Nucleic Acids Research*, 39, W29-W37.

4. Grabherr, M.G., Haas, B.J., Yassour, M., Levin, J.Z., Thompson, D.A., Amit, I. *et al.* (2011). Full-length transcriptome assembly from RNA-Seq data without a reference genome. *Nature Biotechnology*, 29, 644-652.

5. He, S., Johnston, P.R., Kuropka, B., Lokatis, S., Weise, C., Plarre, R. *et al.* (2018). Termite soldiers contribute to social immunity by synthesizing potent oral secretions. *Insect molecular biology*.

6. Krogh, A., Larsson, B., Von Heijne, G. & Sonnhammer, E.L. (2001). Predicting transmembrane protein topology with a hidden Markov model: application to complete genomes. *Journal of Molecular Biology*, 305, 567-580.

7. Petersen, T.N., Brunak, S., von Heijne, G. & Nielsen, H. (2011). SignalP 4.0: discriminating signal peptides from transmembrane regions. *Nature Methods*, 8, 785-786.

8. Rappsilber, J., Mann, M. & Ishihama, Y. (2007). Protocol for micro-purification, enrichment, pre-fractionation and storage of peptides for proteomics using StageTips. *Nature protocols*, 2, 1896.

9. Sury, M.D., McShane, E., Hernandez-Miranda, L.R., Birchmeier, C. & Selbach, M. (2015). Quantitative proteomics reveals dynamic interaction of c-Jun N-terminal kinase (JNK) with RNA transport granule proteins splicing factor proline- and glutamine-rich (Sfpq) and non-POU domain-containing octamer-binding protein (Nono) during neuronal differentiation. *Mol Cell Proteomics*, 14, 50-65.

10. Tyanova, S. & Cox, J. (2018). Perseus: A Bioinformatics Platform for Integrative Analysis of Proteomics Data in Cancer Research. In: *Cancer Systems Biology*. Springer, pp. 133-148.

11. Tyanova, S., Temu, T., Carlson, A., Sinitcyn, P., Mann, M. & Cox, J. (2015). Visualization of LC-MS/MS proteomics data in MaxQuant. *Proteomics*, 15, 1453-1456.

12. Tyanova, S., Temu, T., Sinitcyn, P., Carlson, A., Hein, M.Y., Geiger, T. *et al.* (2016). The Perseus computational platform for comprehensive analysis of (prote)omics data. *Nat Methods*, 13, 731-740.
